# The efficacy of manual toothbrushes in patients with fixed orthodontic appliances: a randomized clinical trial

**DOI:** 10.1186/s12903-023-03035-6

**Published:** 2023-05-23

**Authors:** Fathima Fazrina Farook, Abdulmajeed Alrumi, Khaled Aldalaan, Khansa Ababneh, Abdulsalam Alshammari, Amani Abdullah Al-Khamees, Farraj Albalawi

**Affiliations:** 1 College of Dentistry, King Saud Bin Abdulaziz University for Health Sciences, Riyadh, 11426 Saudi Arabia; 2 King Abdullah International Medical Research Center, Riyadh, Saudi Arabia; 3 Department of Dental Services, Ministry of National Guard-Health Affairs, Riyadh, Saudi Arabia; 4 Department of Dentistry, King Faisal Specialist Hospital and Research Center, Riyadh, Saudi Arabia; 5 College of Dentistry , Majmaah University, Al Majma’ah, Saudi Arabia

**Keywords:** toothbrush, prevention, oral health, plaque control, orthodontic appliances

## Abstract

**Objective:**

This study aims to evaluate three types of manual toothbrushes [Cross action (CA), Flat trim (FT), and orthodontic type (OT)] in terms of efficacy in plaque removal in patients undergoing fixed orthodontic treatment.

**Background:**

Manual toothbrushes are an essential part of oral hygiene for primary prevention. Plaque control, however, can be influenced by a number of individual and material-related factors. Individual factors include the presence of fixed orthodontic appliances on tooth surfaces, such as brackets and bands, which create difficulties with oral hygiene and lead to plaque formation. The evidence for the effectiveness of advanced bristle designs (multilevel, criss-cross) of the manual toothbrush alone in removing plaque in patients undergoing orthodontic therapy is limited.

**Methods:**

The experiment followed the Consolidated Standards of Reporting Trials (CONSORT) guidelines. This was a three treatment, three-period crossover clinical trial with a single brushing exercise. Thirty subjects were randomized to one of the three treatment sequences of different bristle designs: (CA, FT, and OT). The primary outcome measure was the difference in the plaque scores (baseline minus post-brushing) at each study period, as determined by the Turesky-Modified Quigley–Hein Plaque Index.

**Results:**

Of the thirty-four subjects enrolled in the study, thirty of the subjects met the inclusion criteria and completed all three periods of the study. The mean age was 19.5 ± 1.52 years, with a range of 18–23 years. The differences between treatments in plaque score reduction after brushing were statistically significant (*p*-value < .001). The treatment differences were statistically significant (*p*-value < .001) favoring the FT toothbrush over the OT and CA types of toothbrush designs. On the contrary, the difference between the OT and CA types was not statistically significant.

**Conclusions:**

Plaque was significantly removed by the conventional FT toothbrush after a single brushing compared to the OT and CA types.

## Clinical relevance

The OT and the CA toothbrushes are not as effective as the FT toothbrushes in removing dental plaque in participants with fixed orthodontic appliances.

Even though the cross-angled bristle tuft designs appear to work better than the flat or multilevel tuft designs in intact teeth, individual factors such as braces can affect the efficacy of a particular design.

Additional research is required before evidence-based advice concerning the relative performance of the different manual toothbrushes in fixed orthodontic patients can be proven.

## Introduction

Periodontal disease and dental caries are major public health challenges that contribute significantly to the global burden of oral disease [1–3]. Dental biofilm is an important etiologic factor in the inception and development of caries and periodontal disease [4]. The essential role of dental biofilm in the etiology of gingivitis is well established and the removal of dental biofilm can reverse this process [5]. Effective and well-performed dental bio-film control is crucial to enhancing oral health hygiene.

Both mechanical and chemical methods are used to control plaque [6–8]. However, mechanical plaque control is the mainstay of the primary prevention of gingivitis and managing gingivitis as a primary preventive strategy for periodontitis [9]. The most popular mechanical approach for controlling plaque at home is a manual toothbrush, ideally used with fluoride toothpaste [10]. The use of a manual toothbrush plays a fundamental role in oral hygiene for primary prevention [10]. However, a number of individual and material factors contribute to controlling dental plaque accumulation to prevent gingivitis, periodontitis, and decay [11].

Of the individual factors, the presence of a fixed orthodontic appliance on tooth surfaces, such as brackets and bands, creates difficulties in maintaining good oral hygiene, resulting in the build-up of plaque [12]. The brackets, bands, archwires, and elastomeric modules of fixed orthodontic appliances provide additional surface area for bacteria to develop and accelerate the accumulation of plaque and the formation of lesions in areas that would normally have a low risk of caries [13]. The risk is increased when combined with poor oral hygiene [13]. Good oral hygiene is a challenge that patients face during orthodontic treatment because the use of dental floss, interdental brushes, and techniques to brush their teeth requires more effort [14]. Twice daily toothbrushing with interproximal cleaning [15, 16] is recommended as an essential part of a daily plaque control program for all orthodontic patients.

Toothbrush manufacturers target innovations in the brush head design that will support non-ideal tooth brushing techniques and time. One such alteration was the change from the flat toothbrush to multilevel designs by varying the bristle length to improve reaching the interproximal areas [17]. The other development was the angled, rather than the vertical bristle tuft arrangement, which contributed significantly to approximal plaque removal [17–19]. In laboratory and clinical studies with patients with intact teeth, toothbrushes with multilevel profiles were consistently more effective than flat toothbrushes, especially when the interproximal efficacy was monitored. Clinical studies consistently demonstrated that a brush with an angled bristle tuft configuration is significantly more effective [19]. A systematic review by Slot in 2012, indicated that brushes with a multilevel and angled brush head tuft configuration are more efficient than flat trim toothbrushes [17].

In non-orthodontic patients, powered brushes, especially oscillating rotating ones, outperform manual ones in terms of plaque and gingivitis reduction in both short- and long-term [20]. However, for orthodontic appliance users, uncertainty persists. A recent systematic review and meta-analysis concluded that powered and manual toothbrushes do not significantly differ in the reduction of plaque accumulation or gingivitis in patients with fixed orthodontic appliances [21]. The majority of patients preferred a manual toothbrush, conventional or orthodontic, as it is cheaper and easier to use. Evidence of the effectiveness of advanced bristle design (multilevel, criss-cross) of manual toothbrushes in removing plaque in orthodontic patients is limited, with conflicting reports of effectiveness. A few studies compared manual brushes with orthodontic brushes with conflicting reports of effectiveness [16, 22–28]. A recent systematic review and meta-analysis revealed the need for clinical studies to assess the effects of using an orthodontic toothbrush compared to a conventional toothbrush [29]. The current study aimed to clinically compare the effect of three types of manual toothbrushes (CA, FT, and OT brush) on plaque removal when the modified Bass method of brushing is used in patients with fixed orthodontic appliances.

## Methods/study sample

**Fig. 1:**
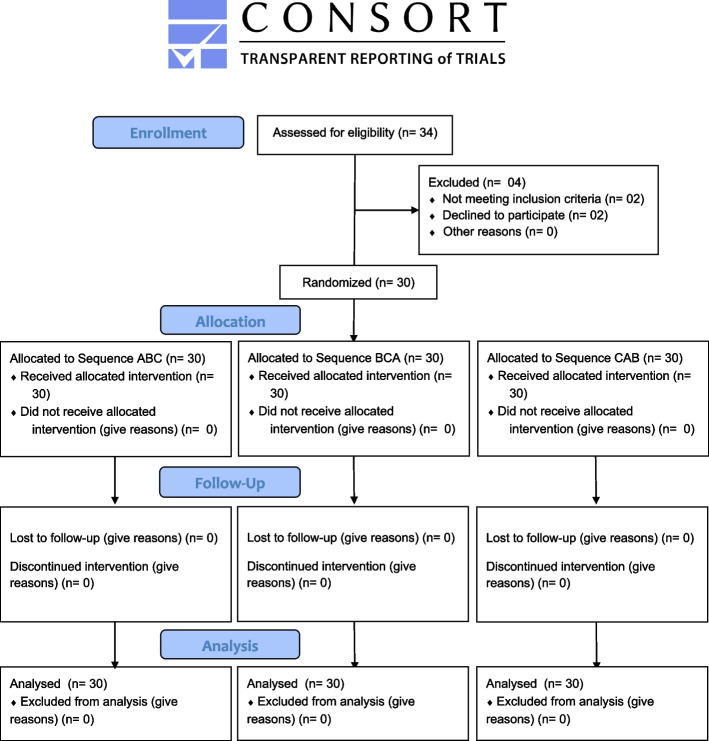
CONSORT flow diagram

The experimental design followed the Consolidated Standards of Reporting Trials (CONSORT) statement (Fig. 1) [30]. This was a randomized 3X3 cross-over, controlled trial, with three arms comparing three different manual brush designs with patients with a fixed orthodontic appliance. The study was approved by the Institutional Review Board and conducted according to the Declaration of Helsinki. The study was conducted from November 2020 to June 2021 and registered in the ISRCTN registry with trial ID ISRCTN14766887.

The participants were from 18 to 25 years, in good general and oral health, and wearing fully bonded conventional metal fixed orthodontic appliances with good hand dexterity without any disabilities. In addition, a minimum of 25 natural teeth with facial and lingual scorable surfaces without any oral lesions or periodontal pockets of 3 mm or loss of attachment/recession of 2 mm was also required. The exclusion criteria were evidence of mucogingival problems, smoking, pregnancy, five or more carious lesions requiring restorative treatment, heavy restorations, or wearing fixed or removable prostheses. Lastly, participation in any other elective dental procedure, including prophylaxis, during the study period or if there was evidence of any disease or condition that may interfere with the study procedures. During the study, participants agreed to refrain from participating in any other oral care study and postpone elective dentistry, including prophylaxis, until the study was completed.

All eligible patients, attending the Orthodontic Clinic at the Dental Centre, Ministry of National Guard—Health Affairs, were approached by the residents and received written study information. They were included in the study only after obtaining written informed consent.

### Sample size estimation

The sample size was determined based on findings in a similar crossover study, [28]. A sample of 30 participants, measured at 3-time points was used to achieve 90% power to detect a difference in the mean using a Geisser-Greenhouse Corrected F Test at a 0.05 significance level. The standard deviation for the participants at the same time point was assumed to be 0.15. The pattern of the covariance matrix required all correlations equal with a correlation of 0.30 between the first and second time point measurements. The standard deviation of the hypothesized mean was 0.05. The calculation was performed in PASS software version 2020 [31].

**Fig. 2:**
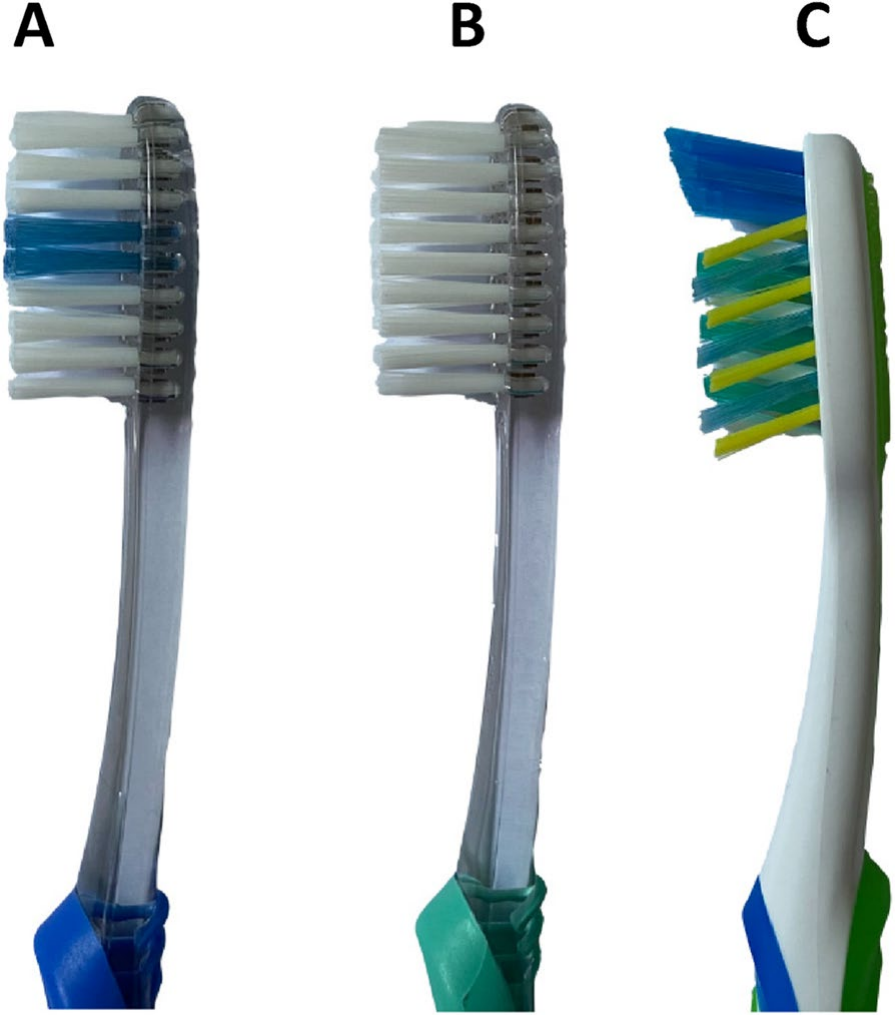
Three different bristle designs of commercially available Oral B manual toothbrushes. **A** Orthodontic brush (Oral-B Pro-Expert Clinic Line Orthodontic Manual Toothbrush). **B** Conventional Oral-B Pro Gum Care Manual Brush. **C** Cross-action design brush (Oral-B Pro-Expert Cross action All In One Manual Toothbrush)

Three different bristle designs of commercially available Oral B manual toothbrushes (CA, OT and FT) were used. The OT brush used was the Oral-B Pro-Expert Clinic Line Orthodontic Manual Toothbrush. The CA design brush used was an Oral-B Pro-Expert Cross action All In One Manual Toothbrush, and the third brush was the FT conventional Oral-B Pro Gum Care Manual Brush. All the brushes were adult brushes with soft brush heads (Fig. 2).

### Randomization (random number generation, allocation concealment, implementation)

The patients were randomly assigned to one of the three treatment sequences determined by a computer-generated plan prepared before the study (ABC, BCA, and CAB) with 30 participants in each treatment sequence. Group allocation and distribution of toothbrushes was done by an investigator who was blinded to the data collection. Allocation concealment was achieved with sequentially numbered, opaque, sealed envelopes containing the treatment allocation cards, which were prepared before the trial.The participants were recalled four times (visit and scored 1, 2, 3, and 4) with a wash-out period of 1 week between each visit.

As part of the study, the participants consented not to brush their teeth the day before each appointment and refrain from eating, drinking, and smoking for 4 h before each visit, except for small sips of water. Two evaluators who were blinded to the group allocation evaluated the plaque. The inter-examiner reliability test for the evaluation of the plaque score was conducted and calculated using the Intraclass correlation coefficient.

The sample’s understanding of the brushing method was evaluated by the investigator before beginning the study. On Day 1, the patients were interviewed, and the relevant information was obtained regarding their demography, and regular oral hygiene behavior and they received a dental clinical examination that included recording the number of teeth present.

On day 1, all the patients received oral and written instructions regarding the use of their assigned toothbrush with the Modified Bass method. The toothbrush is placed at the gum margin at an angle of 45° and making small, vibratory movements, also moving along the dental arches until all accessible tooth surfaces had been brushed. The tooth brushing was performed for 2 min (30 s for each quadrant) with the assigned toothbrush, using standard fluoride toothpaste in front of the examiner during each appointment. After brushing, the participants were instructed to lightly rinse their mouths once with water.

During each visit, the plaque was recorded using a red disclosing solution to obtain the baseline data. Plaque assessments were performed by two periodontists who were blinded to the brushing method, using the Turesky Modified Quigley-Hein Plaque Index [32, 33]. The scores were re-evaluated by the same examiners independently after brushing and swishing with disclosing solution again. The examiners received prior training with the method.

The same procedure was done during subsequent appointments using the other toothbrushes that were randomly assigned to each participant. All three periods followed the same procedure. Participants' accountability was documented at Period 3.

The criteria for plaque scoring using the Turesky Modified Quigley-Hein Plaque Index, were as follows [32].

**Table:** 

0	no plaque
1	separate flecks of plaque present at the cervical margin
2	continuous band of plaque up to 1 mm at the cervical margin
3	a band of plaque that is wider than 1 mm but covering less than one-third of the side of the crown of the tooth
4	plaque covering at least one-third but less than two-thirds of the side of the crown
5	plaque covering more than two-thirds of the side of the crown

The buccal and lingual surfaces of each tooth were scored. A total of 40 surfaces on 20 teeth were scored per participant. The average values for each participant were calculated by dividing the total score by the number of teeth examined.

### Outcomes (primary and secondary) and any changes after trial commencement

A whole-mouth average score was obtained by averaging the plaque scores of each participant at baseline (before brushing) and after brushing. Our outcome (for each participant in each treatment period) was the difference between baseline and post-brushing scores. Both self-reported non-serious oral-related adverse events and all serious adverse events were recorded in the study on the appropriate case report form. Only incidents of whole-body adverse effects were collected if they were likely associated with the product.

### Blinding

Blinding of the patients was not possible. Plaque assessments were performed by two periodontists who were blinded to the treatment groups.

### Statistical analyses

Descriptive statistics were used to summarize the sample characteristics and baseline dental measurements. A mean and standard deviation (SD) were calculated for the three groups at baseline and after brushing. To analyze the effect of the type of toothbrush on the plaque, a mixed-effects linear model was used to account for the repeated measurements that yield period, sequence, intra-patient, and inter-patient variability.

An analysis of differences between treatment groups was performed using a mixed model for repeated measures on the difference scores (baseline minus post-brushing) for each participant, for each period. The factors in the model were subject (random effect), period, treatment, carryover, and baseline whole-mouth average scores as covariates. A carryover not reaching the significance level of 10% was deleted from the final statistical model. The Intraclass correlation (ICC) evaluated the consistency or reliability of the two examiners with respect to the average pre-brushing and post-brushing results.

NCSS software (Version 20) ® was used for data entry and analysis [34]. *P* values < 0.05 were considered significant.

## Results

### Participants flow

Thirty (30) participants enrolled in the trial were randomized to one of the three treatment sequences, and completed all visits. In total, 34 participants were entered into the study (Fig. 1- CONSORT Flow Diagram).

### Baseline data

The patient characteristics and baseline plaque scores are summarized in Table 1. The mean age was 19.5 ± 1.52 years, with a range of 18–23 years. The majority (63.3%, *n* = 19) of the participants were female (Table 1).

**Table 1:** Demographic characteristics of the sample

Characteristics	Mean ± SD	Min	Max
Age	19.5 ± 1.52	18	23
Gender	**N (%)**		
Female	19 (63.3)		
Male	11 (36.7)		

### Analysis for the outcome, estimation and precision, subgroup analyses

The F-test for the treatment was significant (*F*-value = 7.65, *p*<0.001) with no period effect (*F*-value = 2.23, *p*=0.117) and no carryover effect (*F*-value = 2.129,  *p*=0.241). The treatment differences were statistically significant between the flat trim and orthodontic brush groups (0.3178, *p*-value < 0.001) favoring the flat trim toothbrush over the orthodontic brush (*p* < 0.001) and between the FT and CA (*p* = 0.004) toothbrush, favoring the flat trim. However, the difference between the OT and CA types was not statistically significant (*p* = 0.511). The ICC analysis showed a high degree of consistency between the two examiners, pre-brushing (ICC ≥ 0.978; *p* < 0.001) and post brushing (ICC ≥ 0.983; *p* < 0.001). These results indicate a high degree of consistency or reliability between the two examiners (Table 2).

**Table 2:** Mixed model analysis

**Treatment**	***B*** **(SE of** ***B*)**	***β*** **(95% confidence limits)**	***P*** **value**
Conventional flat trim	0.26 (0.088)	0.08–0.44	< 0.001
Orthodontic	-0.06 (0.087)	-0.23–0.12	0.5118
Crossaction	*Ref*		
**Individual Comparison Hypothesis Test Results**			
	Comparison (mean difference)	F-value	Adjusted *p*-value
Conventional flat trim vs Orthodontic	0.32	13.3341	<0.001
Conventional flat trim vs Crossaction	0.26	8.6889	0.004
Orthodontic vs Crossaction	-0.06	0.4360	0.511

The Intragroup comparisons for the three groups showed a statistically significant increase in plaque elimination in the anterior and posterior groups of teeth, with the FT group (*p* < 0.001). No other significant change was found for the other tooth groups. The subgroup analysis was done for the anterior and posterior teeth, and demonstrated a significant effect for the FT brush over the OT and CA brushes (anterior, posterior). The difference between the OT and CA types was not statistically significant for the anterior and posterior sides.

Harms no serious harm was observed.

## Discussion

This study was conducted to assess the effectiveness of three manual toothbrushes (CA, FT, and OT) on plaque removal by using the Modified Bass method of brushing in patients who had fixed orthodontic appliances. The FT toothbrush was significantly better at reducing plaque than the OT and CA toothbrushes. No differences were found between the OT brush and CA brushes. Our results revealed, the FT performed better than the OT and CA brushes in patients with fixed orthodontic appliances.

There are many toothbrushes specifically designed for patients with braces. The manual orthodontic toothbrush is one of several hygiene tools that orthodontists recommend to their patients [34]. It is important to know if the product is superior compared to the conventional standard manual FT toothbrush and the CA type.

The majority of patients preferred utilizing the orthodontic toothbrush because of its unique bristle design, which made cleaning the area around bonded brackets easier. Studies on the effectiveness of OT toothbrushes report conflicting results. According to some studies, FT and OT toothbrushes are not significantly different [23, 25, 27, 35]. The orthodontic toothbrush was shown in one study to reduce plaque significantly [27]. Based on the baseline and follow-up plaque indexes from another study, there was a significant difference between the two groups for the orthodontic toothbrush Although some studies did not find that orthodontic toothbrushes reduced plaque as effectively as conventional toothbrushes, there were no reports of worsening oral health following the re-evaluation [23, 36]. Some studies reported statistically significant differences for the anterior and posterior aspects of the individual tooth groups. One study observed a greater efficacy of the orthodontic toothbrushes, and this difference was limited to the anterior region of the mouth, and considered by the authors of the original study to be of small clinical significance [24]. Kiliçoğlu et al. [25] reported a statistically significant increase in plaque elimination in specific teeth, namely the upper premolars and lower anterior teeth with the orthodontic toothbrush.

Our study found significant plaque reduction in the anterior and posterior tooth groups with the FT toothbrush, compared with the OT, and CA toothbrushes. Our findings are also slightly different from the findings from a recent meta-analysis which showed that the OT toothbrushes resulted in a greater reduction in plaque, compared to the conventional toothbrush [29]. However, the effectiveness in reducing gingival bleeding in the OT toothbrush is questionable.

The reasons for this could be explained by the fact that certain bristle configurations, like lowered bristles in the middle of the brush field, may have had lower cleaning effectiveness compared to planar bristle fields around the gingival sulcus area, possibly because the brush ends did not engage with the gingival sulcus to remove plaque buildup. In addition, depending on the force used to use them, some toothbrushes have a variable cleaning effect. There are questions about the toothbrush's efficiency in cleaning the gingiva, despite assertions that its V-shaped bristles increase contact with orthodontic appliance.

The use of a toothbrush with an angled bristle tuft configuration has consistently been proven more effective in clinical studies with intact teeth [18, 19]. A systematic review by Slot in 2012, revealed that brushes with an angled brush head tuft configuration are more efficient than flat-trim toothbrushes. Cross-angled bristle tuft designs appear to work better than flat or multilevel tuft designs [17]. The difference in performance between orthodontic and non-orthodontic patients may be due to the higher plaque retention effects of fixed appliances. Cross-action toothbrushes might be less effective in cleaning the area surrounding orthodontic braces than smooth surfaces because smooth surfaces are simpler to keep clean. The angled bristles may become entangled around the bracket, diminishing cleaning efficacy.

### Strengths

Several advantages of our study include its crossover design, which serves as a control since patients serve as their own. In addition to reducing confounders such as age, gender, and hand skills among participants, it is an ideal model to estimate the period, carryover, and treatment effects.

Our methodology is another strength. The brushing technique was standardized to Modified Bass, and the brushing time was standardized to 2 min, as recommended by many orthodontists [37]. In addition to standardized hygiene instructions, the toothpaste we used did not contain any additives which could modify plaque accumulation. As a result, the influence of these products on these groups was avoided. In addition, the standardization of the hygiene instructions. Some studies adopted their instructions [24, 27]. Another study used the scrubbing backward and forwards method [26], and another the Modified Bass technique [23, 25, 36].

Lastly, our study was short-term, single-use trials. Short-term trials are useful in controlling confounding variables, for example, participant compliance.

### Limitations

One of the major limitations of our study was that no long-term effects could be assessed since treatments were performed as a single exercise. We could not report any adverse effects that could be related to more prolonged use of the toothbrushes. RCTs designed to compare the short and long-term effects of different manual toothbrushes are needed to increase the confidence of these findings, which are very low to moderate. Despite our finding that the FT toothbrush is superior in preventing plaque, we did not find that it was better at controlling gingival bleeding. In other words, other aspects such as gingivitis were not assessed. Even though the patients in the study were not shown the product packing or given any additional information about the toothbrushes being tested, they were able to easily distinguish between the different types of toothbrushes. In this light, it is difficult to determine if the statistically significant difference in plaque reduction was due to the FT toothbrush or a placebo effect.

The results of this cross-over RCT demonstrated that the OT and the CA toothbrushes are not as effective as the FT toothbrush in removing dental plaque in participants with fixed orthodontic appliances. This indicates that even though the cross-angled bristle tuft designs appear to work better than the flat or multilevel tuft designs in intact teeth [17], individual factors such as braces can affect the efficacy of a particular design [11]. The level of evidence for the efficacy of the orthodontic toothbrush in orthodontic patients is minimal [29]. At this level, the superiority of any bristle design is yet to be established. Considering the limitations of this study, additional research is required before evidence-based advice concerning the relative performance of the different manual toothbrushes in fixed orthodontic patients can be proven.

## Conclusion

Within the limits of this study, the FT toothbrush removed significantly more plaque after a single brushing than the OT and CA types. The FT toothbrush tested in this study was effective in removing dental plaque in patients wearing fixed orthodontic appliances.

## Data Availability

The datasets used and analyzed during the current study are not publicly available as it could compromise research participant consent and is available from the corresponding author on reasonable request.
